# Supplementary material for: CVAPPS: A Cross-Sectional Study of SARS-CoV-2 Vaccine Acceptance, Perceptions, and Post-Vaccination Side Effects among Rheumatic Disease Patients in Kuwait
Source: Vaccines (Basel). 2023 Mar 15;11(3):666. doi: 10.3390/vaccines11030666 (PMC10054305; doi:10.3390/vaccines11030666)
Supplement: Supplementary file 1 [file vaccines-11-00666-s001.zip › vaccines-2239066-supplementary/Supplementary file, Arabic.pdf]

عزيزي المريض

نتمنى أن تصلك تلك الرسالة وأنت في أفضل حال.
انطلاقًا من رؤية ومنظور المسئولية الصحية للرابطة الكويتية لأطباء الروماتيزم، فإننا نقدم إليكم هذا الاستبيان الذي يُلقي الضوء على تلقي لقاح كوفيد-19، والذي نهدف من خلاله إلى ما يلي:

- ✓ تقدير الأعداد النسبية للمرضى الذين تلقوا لقاح كوفيد-19 من بين المرضى الذين يعانون من الأمراض الروماتيزمية.
- ✓ دراسة الأعراض قصيرة الأجل التي تلت تلقِّي اللقاح، وما إذا كان قد حدث أي احتدام للمرض الروماتيزمي.
- ✓ تسليط الضوء على الأسباب الحقيقية وراء امتناع الأشخاص عن تلقي اللقاح (الأسباب الأكثر شيوعًا للامتناع عن تلقي اللقاح).

نتمنى للجميع السلامة، ونشكركم على مشاركتكم في ملء هذا الاستبيان.

معًا سنقضي على الجائحة.

مع خالص التحية،
الرابطة الكويتية لأطباء الروماتيزم

تعد هذه الوثيقة العربية للنص الهدف ترجمة دقيقة للوثيقة الإنجليزية للنص المصدر، حررت في 13 يونيو 2021 بواسطة شركة فارما- ميد للترجمة الطبية المعتمدة، ج.م.ع. ، بريد إلكتروني: orders@pharmamed-eg.com
جميع الحقوق محفوظة.
PHARMA MED Accreditation No.11271
ISO 17100 LICS Language Industry Certification System

أهلية المشاركة في الاستبيان الخاص بتلقي لقاح كوفيد-19

كيف علمت بهذا الاستبيان؟

- ❑ طبيب الروماتيزم
- ❑ الممرضة في غرفة التسريب
- ❑ وسائل التواصل الاجتماعي
- ❑ شركة أدوية
- ❑ أحد الأصدقاء/الأقرباء
- ❑ طرق أخرى

أهلية المشاركة في الاستبيان الخاص بتلقي لقاح كوفيد-19:

- ❑ أن تكون مُشخصًا بأنك تعاني من مرض روماتيزمي
- ❑ أن تكون بالغًا من العمر 21 عامًا أو أكثر.

ملحوظة: أنت مؤهل لاستكمال الاستبيان سواء كنت قد تلقيت لقاح كوفيد-19 أم لا.

الإقرار بالموافقة

من خلال المشاركة، فأنا أؤكد أني أبلغ من العمر 21 عامًا على الأقل وأوافق على استخدام المعلومات التي أقدمها في التحليل الذي تقوم به الرابطة الكويتية لأطباء الروماتيزم.
وأفهم أن بياناتي ستكون مجهولة الهوية، ولن يكون من الممكن تحديد هويتي الشخصية من خلال الإجابات التي سأقدمها اليوم.
وأوافق للرابطة الكويتية لأطباء الروماتيزم على مشاركة بيانات الاستبيان (بدون الإفصاح عن هويتي) لأغراض تتعلق بالتوعية المجتمعية والتعليم والأبحاث العلمية.

- ❑ تأكيد
- ❑ الخروج من الاستبيان

تخطٍّ إلى: إنهاء الاستبيان إذا اختار المشارك "الخروج من الاستبيان"

تعد هذه الوثيقة العربية للنص الهدف ترجمة دقيقة للوثيقة الإنجليزية للنص المصدر، حررت في 13 يونيو 2021 بواسطة شركة فارما- ميد للترجمة الطبية المعتمدة، ج.م.ع. ، بريد إلكتروني: orders@pharmamed-eg.com
جميع الحقوق محفوظة.
PHARMA MED Accreditation No.11271
LICS ISO 17100 Language Industry Certification System

1. الخصائص الديموجرافية

1.1. في أي عام وُلدت؟

تخطٍّ إلى: إنهاء الاستبيان إذا كان الشرط: في أي عام وُلدت؟ أكبر من أو يساوي 2004، تخطٍّ إلى: إنهاء الاستبيان.

1.2. ما نوعك؟

- ❑ أنثى
- ❑ ذكر

1.3. ما جنسيتك؟

قائمة منسدلة بها كل الدول

1.4. في أي دولة تعيش؟

قائمة منسدلة بها كل الدول

1.5. من طبيب الروماتيزم المتابع لحالتك؟

1.6. ما مستواك التعليمي؟

- ❑ شهادة التعليم الابتدائي
- ❑ شهادة التعليم المتوسط
- ❑ شهادة التعليم العالي
- ❑ شهادة مدرسة عليا مهنية
- ❑ درجة البكالوريوس
- ❑ درجة الماجستير
- ❑ درجة الدكتوراه
- ❑ أخرى (...........)

1.7. هل أنت مقدم رعاية صحية؟

- ❑نعم
- ❑لا

تعد هذه الوثيقة العربية للنص الهدف ترجمة دقيقة للوثيقة الإنجليزية للنص المصدر، حررت في 13 يونيو 2021 بواسطة شركة فارما- ميد للترجمة الطبية المعتمدة، ج.م.ع. ، بريد إلكتروني: orders@pharmamed-eg.com
جميع الحقوق محفوظة.

1.8. ما وضعك المهني أو الوظيفي؟

- ❑ موظف/أعمل
- ❑ لست موظفًا/لا أعمل
- ❑ متقاعد
- ❑ طالب
- ❑ غير ذلك (يُرجى التحديد)

1.9. ما حالتك الاجتماعية؟

- ❑ أعزب
- ❑ متزوج
- ❑ مطلق/أرمل

2. الوضع الطبي

2.1. هل تدخن؟

- ❑ نعم - أدخن حاليًا
- ❑ لا - كنت أدخن؛ ولكني أقلعت عن التدخين
- ❑ لا - لم أدخن أبدًا

2.2. ما المرض الذي شُخصت بأنك مصاب به مما يلي؟ [اختر خيارًا واحدًا]

❑التهاب المفاصل الروماتويدي.
❑الذِّئْبَة الجهازية
❑التصلب الجهازي
❑التهاب الفقار اللاصق
❑التهاب المفاصل الصدفي
❑التهاب الأوعية الدموية
❑الالتهاب الجلدي العضلي/التهاب العضلات المتعدد
❑داء بهجت
❑مرض النقرس/النقرس الكاذب
❑متلازمة شوغرن
❑التهاب المفاصل التفاعلي
❑التهاب المفاصل المصاحب لداء الأمعاء الالتهابي
❑مرض الأنسجة الضامة
❑التهاب المفاصل لدى الصغار
❑الألم العضلي الليفي
❑التهاب المفاصل غير المتمايز
❑داء ستيل (مرض التهابي)
❑لم أُشخص بأني مصاب بمرض روماتيزمي
❑غير متأكد
غير ذلك [يُرجى التحديد] ....

تخطٍّ إلى: إنهاء الاستبيان إذا تم تشخيصك بأي مما يلي؟ = لم أُشخص بأني مصاب بمرض روماتيزمي

تعد هذه الوثيقة العربية للنص الهدف ترجمة دقيقة للوثيقة الإنجليزية للنص المصدر، حررت في 13 يونيو 2021 بواسطة شركة فارما- ميد للترجمة الطبية المعتمدة، ج.م.ع. ، بريد إلكتروني: orders@pharmamed-eg.com
جميع الحقوق محفوظة.
PHARMA MED Accreditation No.11271
ISO 17100 LICS Language Industry Certification System

2.3. في أي عام تقريبًا شُخصت بأنك مصاب بحالة من مرض روماتيزمي؟
على سبيل المثال: 1996

2.4. أي أدوية مما يلي كنت تتناولها في الأشهر الثلاثة الماضية لعلاج المرض الروماتيزمي؟ [اختر كل ما ينطبق]

❑أباتاسيبت (أورينسيا)
❑أداليموماب (هوميرا)
❑أبريميلاست (أوتيزلا)
❑أزاثيوبرين (إميوران)
❑باريسيتينيب (الوميانت)
❑ بيليموماب (بنليستا)
❑سيرتيزولوماب (سيمزيا)
❑كلوروكين (دافوكين)
❑الكورتيكوستيرويدات
❑سيكلوفوسفاميد
❑إيتانيرسيبت (انبريل)
❑جوليموماب (سيمبوني)
❑هيدروكسي كلوروكين (بلاكونيل)
❑إنفليكسيماب (ريميكيد)
❑لفلونوميد (ارافا)
❑ميثوتريكسات
❑ميكوفينوليت موفيتيل (سيلسبت/مايفورتيك)
❑ريتوكسيماب (مابثيرا)
❑سيكوكينوماب (كوزنتكس)
❑سالفاسالازين (سالازوبيرين)
❑تاكروليموس (بروجراف)
❑توسيليزوماب (أكتيمرا)
❑توفاسيتينيب (زيلجانز)
❑أوستيكينوماب (ستيلارا)
❑لا أتناول أي أدوية
❑غير متأكد
❑غير ذلك [يُرجى التحديد] ....

تعد هذه الوثيقة العربية للنص الهدف ترجمة دقيقة للوثيقة الإنجليزية للنص المصدر، حررت في 13 يونيو 2021 بواسطة شركة فارما- ميد للترجمة الطبية المعتمدة، ج.م.ع. ، بريد إلكتروني: orders@pharmamed-eg.com
جميع الحقوق محفوظة.
PHARMA MED Accreditation No.11271
LICS Language Industry Certification System ISO 17100

2.5. ما أفضل وصف يمكن أن تصف به مرضك الروماتيزمي في الثلاثة أشهر السابقة؟

❑غير نشط

❑نشط بدرجة بسيطة إلى معتدلة

❑نشط بدرجة شديدة

2.6. هل تعاني من أي أمراض مزمنة أخرى؟

❑ مرض السُّكَّرِي.

❑ مرض بالقلب

❑ ارتفاع ضغط الدم

❑ مرض بالرئة

❑ السمنة

❑ مرض بالكُلى

❑ السرطان

❑ نقص المناعة، بما في ذلك فيروس نقص المناعة البشرية

❑ لا يوجد

❑ غير ذلك [يُرجى التحديد]

تعد هذه الوثيقة العربية للنص الهدف ترجمة دقيقة للوثيقة الإنجليزية للنص المصدر، حررت في 13 يونيو 2021 بواسطة شركة فارما- ميد للترجمة الطبية المعتمدة، ج.م.ع. ، بريد إلكتروني: orders@pharmamed-eg.com
جميع الحقوق محفوظة.

PHARMA MED
Accreditation No.11271

ISO 17100 LICS Language Industry Certification System

3. الوضع المتعلق بمرض كوفيد-19

3.1. هل أجريت مسحة طبية وكانت النتيجة إيجابية وتؤكد أنك مصاب بمرض كوفيد-19؟

- ❑ نعم
- ❑ لا

إذا كانت الإجابة "لا"

3.1.1. هل خضعت للحجر الصحي خلال الفترة من 2019 إلى 2021؟

- ❑ نعم
- ❑ لا

إذا كانت الإجابة "نعم"

3.1.1.1. ماذا كان السبب؟

- ❑ السفر
- ❑ مخالطة شخص مصاب بالفيروس

3.1.2. إلى أي مدى تقلق من التعرض للإصابة بمرض كوفيد-19؟

- ❑ لا أقلق
- ❑ أقلق بدرجة بسيطة
- ❑ أقلق بدرجة متوسطة
- ❑ أقلق بدرجة شديدة
- ❑ أقلق بدرجة شديدة للغاية

3.1.3. إلى أي مدى تقلق من الإصابة بعدوى كوفيد-19 بدرجة شديدة؟

- ❑ لا أقلق
- ❑ أقلق بدرجة بسيطة
- ❑ أقلق بدرجة متوسطة
- ❑ أقلق بدرجة شديدة
- ❑ أقلق بدرجة شديدة للغاية

إذا كانت الإجابة "نعم"

3.2.1. في أي تاريخ كانت نتائج التشخيص إيجابية؟ [أدخل التقويم هنا]

3.2.2. كيف سارت الأمور؟ [اختر خيارًا واحدًا]

- ❑ لم أودع بالمستشفى
- ❑ أودعت بالمستشفى ولكني لم أوضع على جهاز الأكسجين
- ❑ أودعت بالمستشفى ووضعت على جهاز الأكسجين
- ❑ أودعت بالمستشفى بقسم العناية المركزة

تعد هذه الوثيقة العربية للنص الهدف ترجمة دقيقة للوثيقة الإنجليزية للنص المصدر، حررت في 13 يونيو 2021 بواسطة شركة فارما- ميد للترجمة الطبية المعتمدة، ج.م.ع. ، بريد إلكتروني: orders@pharmamed-eg.com
جميع الحقوق محفوظة.
PHARMA MED Accreditation No.11271
ISO 17100 LICS Language Industry Certification System

3.3. منذ أن بدأت الجائحة، هل تعرض أي شخص من المحيطين بك (من الأصدقاء أو الزملاء أو الأقرباء) للإصابة بعدوى كوفيد بدرجة شديدة (استلزمت إيداعه بالمستشفى أو دخوله إلى قسم العناية المركزة أو أدت إلى وفاته)؟

- ❑ نعم
- ❑ لا

تعد هذه الوثيقة العربية للنص الهدف ترجمة دقيقة للوثيقة الإنجليزية للنص المصدر، حررت في 13 يونيو 2021 بواسطة شركة فارما- ميد للترجمة الطبية المعتمدة، ج.م.ع. ، بريد إلكتروني: orders@pharmamed-eg.com
جميع الحقوق محفوظة.

4. الوضع المتعلق باللقاح

4.1. هل تعاني من حساسية تجاه أي لقاح؟

- ❑ نعم
- ❑ لا
- ❑ غير متأكد/لا أتذكر

4.2. هل رفضت من قبل أن تتلقى لقاحًا موصى به أو تعطيه لأحد أطفالك؟

- ❑ نعم
- ❑ لا
- ❑ غير متأكد/لا أتذكر

4.3. هل تلقيت لقاح الإنفلونزا (الوقاية من نزلات البرد) في عام 2019؟

- ❑ نعم
- ❑ لا
- ❑ غير متأكد/لا أتذكر

4.4. هل تلقيت لقاح الإنفلونزا (الوقاية من نزلات البرد) في عام 2020؟

- ❑ نعم
- ❑ لا
- ❑ غير متأكد/لا أتذكر

4.5. هل تلقيت لقاح المكورات الرئوية؟

- ❑ نعم
- ❑ لا
- ❑ غير متأكد/لا أتذكر

تعد هذه الوثيقة العربية للنص الهدف ترجمة دقيقة للوثيقة الإنجليزية للنص المصدر، حررت في 13 يونيو 2021 بواسطة شركة فارما- ميد للترجمة الطبية المعتمدة، ج.م.ع. ، بريد إلكتروني: orders@pharmamed-eg.com
جميع الحقوق محفوظة.

PHARMA MED Accreditation No.11271
AUTHORIZED BY EGYPTIAN TRANSLATORS ASSOCIATION
EGYPTIAN & INTERNATIONAL FEDERATION OF TRANSLATORS (FIT) AND UNESCO
TO PRODUCE & PROOFREAD TRANSLATION WORKS IN All Languages

ISO 17100 LICS Language Industry Certification System

5. لقاح كوفيد-19

5.1. هل استشرت طبيبك بخصوص لقاح كوفيد-19؟

- ❑ نعم
- ❑ لا
- ❑ غير متأكد/لا أتذكر

5.2. هل نصحك الطبيب بعدم تلقي اللقاح؟

- ❑ نعم
- ❑ لا
- ❑ غير متأكد/لا أتذكر

5.3. هل تطالع صحيفة النصائح عبر الإنترنت التي تقدمها الرابطة الكويتية لأطباء الروماتيزم بخصوص لقاح كوفيد-19؟

- ❑ نعم
- ❑ لا
- ❑ غير متأكد/لا أتذكر

5.4. ما المصدر الذي تتلقى منه معلوماتك عن اللقاح؟ (اختر كل ما ينطبق)

- ❑ ليست لدي معلومات
- ❑ وسائل الأخبار (التلفاز/الراديو/الجرائد)
- ❑ وسائل التواصل الاجتماعي
- ❑ نصائح الصحة العامة التي تقدمها الحكومة
- ❑ الأصدقاء/العائلة
- ❑ المنشورات الطبية
- ❑ طبيبك (أطباؤك) أو أخصائيو الرعاية الصحية الآخرون
- ❑ المريض أو مؤسسة (مؤسسات) مهنية
- ❑ غير ذلك (يُرجى التحديد)

تعد هذه الوثيقة العربية للنص الهدف ترجمة دقيقة للوثيقة الإنجليزية للنص المصدر، حررت في 13 يونيو 2021 بواسطة شركة فارما- ميد للترجمة الطبية المعتمدة، ج.م.ع. ، بريد إلكتروني: orders@pharmamed-eg.com
جميع الحقوق محفوظة.
PHARMA MED Accreditation No.11271
ISO 17100 LICS Language Industry Certification System

5.5 هل تلقيت لقاح كوفيد-19؟

❑ نعم

❑ لا

إذا كانت الإجابة "نعم":

5.5.1. ما كان السبب لتلقيك لقاح كوفيد-19؟

❑ لأني أخشى الإصابة/الإصابة بدرجة شديدة بعدوى كوفيد-19

❑ لأني أخشى أن أتوفى متأثرًا بإصابتي بكوفيد-19

❑ لأني أرغب في حماية أسرتي من الإصابة بكوفيد-19

❑ لأن تلقي اللقاح سيُقلل الأعباء المالية التي أتكبدها بسبب الجائحة

❑ لأني أعتقد أن اللقاح هو الوسيلة الوحيدة التي يمكن أن تساعدنا على أن تعود حياتنا لما كانت عليه قبل تفشي كوفيد-19

❑ لأني أثق في العموم في المعلومات والتعليمات التي تصدر عن الحكومة

5.5.2. ما اللقاح الذي تلقيته؟

❑ فايزر/بيوأنتك

❑ أسترازينيكا/أوكسفورد

❑ مودرنا

❑ جونسون آند جونسون

❑ سبوتنيك V

❑ سينوفاك

❑ غير ذلك (يُرجى التحديد)

5.5.3. متى كانت الجرعة الأولى؟

[أدخل التقويم هنا]

5.5.4. متى كانت الجرعة الثانية؟

(اتركها فارغة إذا لم تكن قد تلقيتها بعد أو إذا كانت غير قابلة للتطبيق)

[أدخل التقويم هنا]

5.5.5. هل توقفت عن تناول أدويتك أو أجريت عليها أي تعديلات؟

❑ نعم

❑ لا

❑ غير متأكد/لا أتذكر

5.5.6. إذا كانت الإجابة "نعم"، فيُرجى التحديد [..........]

تعد هذه الوثيقة العربية للنص الهدف ترجمة دقيقة للوثيقة الإنجليزية للنص المصدر، حررت في 13 يونيو 2021 بواسطة شركة فارما- ميد للترجمة الطبية المعتمدة، ج.م.ع. ، بريد إلكتروني: orders@pharmamed-eg.com
جميع الحقوق محفوظة.
PHARMA MED Accreditation No.11271
LICS ISO 17100 Language Industry Certification System

5.5.6. هل تعرضت لأي آثار جانبية بعدما تلقيت لقاح كوفيد- 19 الخاص بك؟

- ❑ التَّأق
- ❑ جلطات دموية
- ❑ اضطرابات بضغط الدم
- ❑ اضطرابات بمستوى السكر في الدم
- ❑ ألم/وجع بالجسم
- ❑ طفح جلدي بالوجه والجسم
- ❑ إرهاق
- ❑ حمّى
- ❑ صداع
- ❑ تورم المفاصل
- ❑ كدمات/طفح جلدي موضعي في موضع الحقن
- ❑ غثيان/قيء
- ❑ أعراض عصبية
- ❑ ألم في موضع الحقن
- ❑ صعوبة في التَّنَفُّس
- ❑ احتدام تفاقم المرض الروماتيزمي (تطلب الذهاب إلى الطبيب)
- ❑ تورم العقد الليمفاوية
- ❑ لا يوجد
- ❑ غير ذلك (يُرجى التحديد)

5.5.7. كم من الوقت استمرت الآثار الجانبية؟

- ❑ من 1 إلى 5 أيام
- ❑ من 5 إلى 10 أيام
- ❑ من 10 إلى 14 يومًا
- ❑ أكثر من 14 يومًا

5.5.8. هل تعتقد أن تلقي لقاح كوفيد-19 يجب أن يكون إلزاميًّا؟

- ❑ نعم
- ❑ لا

إذا كانت الإجابة "لا":

5.5.9. لم تتلقَّ لقاح كوفيد-19 للسبب الآتي:

❑ لأني مازلت أنتظر تلقي اللقاح

❑ لأني أعارض تلقي لقاح كوفيد-19

❑ لأني طلبت تحديد موعد؛ ولكن وزارة الصحة رفضته (بسبب معاناتي من التأق أو لأني حامل أو لأني أصبت بكوفيد-19 في آخر 90 يومًا أو لأني أصغر من 16 عامًا أو لأني أعاني من عدوى نشطة)

تخطٍّ إلى "القسم 6" إذا لم تتلقَّ لقاح كوفيد-19 للسبب الآتي = لأني أعارض حملة تلقي لقاح كوفيد-19

تعد هذه الوثيقة العربية للنص الهدف ترجمة دقيقة للوثيقة الإنجليزية للنص المصدر، حررت في 13 يونيو 2021 بواسطة شركة فارما- ميد للترجمة الطبية المعتمدة، ج.م.ع. ، بريد إلكتروني: orders@pharmamed-eg.com
جميع الحقوق محفوظة.
PHARMA MED Accreditation No.11271
LICS ISO 17100 Language Industry Certification System

6. معارضة تلقي اللقاح

فقط للمشاركين الذين كانت إجابتهم عن السؤال "لم تتلقَّ لقاح كوفيد-19 للسبب الآتي" = لأني أعارض حملة تلقي لقاح كوفيد-19

6.1. ما أفضل عبارة تصفك مما يلي؟

- ❑ أعارض تلقي اللقاح بشكل عام
- ❑ أعارض تلقي لقاح كوفيد-19 لأني غير متأكد من فعَّاليته
- ❑ أعارض تلقي لقاح كوفيد-19 لأني قلق من آثاره الجانبية
- ❑ أعارض تلقي لقاح كوفيد-19 لعدم توفر بيانات كافية، ولأنه قد تم استبعاد المرضى المصابين بمرض روماتيزمي من الدراسات
- ❑ أعتقد أني قد أُصاب بعدوى كوفيد-19 بعد تلقي اللقاح
- ❑ أعارض تلقي لقاح كوفيد-19 لأني أخشى أن يفاقم مرضي أو يتعارض مع علاجي
- ❑ أعارض تلقي لقاح كوفيد-19 لأني أعتقد أنه يُوصى به لأسباب اقتصادية بدعم من شركات الأدوية
- ❑ السبب وراء رفضي لتلقي اللقاح هو لأني مصاب برهاب (فوبيا) الحقن
- ❑ السبب وراء رفضي لتلقي اللقاح هو لأني قد عانيت سابقًا من تأق للقاح

6.2. هل سترغب في تلقي اللقاح إذا توفرت بيانات أكثر عن أمان وفعالية اللقاحات المختلفة؟

- ❑ نعم
- ❑ لا
- ❑ لا أعرف

6.3. هل تلقى أي فرد من أفراد أسرتك (من الدرجة الأولى) اللقاح؟

- ❑ نعم
- ❑ لا
- ❑ لا أعرف

6.4. هل تنصح أيًا من أفراد أسرتك أو أقاربك بألا يتلقوا لقاح كوفيد-19؟

- ❑ نعم
- ❑ لا

6.5. إذا كان تلقي لقاح كوفيد-19 إلزاميًا، فهل ستتلقى اللقاح؟

- ❑ نعم
- ❑ لا
- ❑ لا أعرف

6.6. إذا وُجِّهت إليك دعوة لحضور فعالية لزيادة الوعي بأهمية لقاح كوفيد-19، فهل ستحضر؟

- ❑ نعم
- ❑ لا

تعد هذه الوثيقة العربية للنص الهدف ترجمة دقيقة للوثيقة الإنجليزية للنص المصدر، حررت في 13 يونيو 2021 بواسطة شركة فارما- ميد للترجمة الطبية المعتمدة، ج.م.ع. ، بريد إلكتروني: orders@pharmamed-eg.com
جميع الحقوق محفوظة.
PHARMA MED Accreditation No.11271
ISO 17100 LICS Language Industry Certification System
