# Supplementary material for: CVAPPS: A Cross-Sectional Study of SARS-CoV-2 Vaccine Acceptance, Perceptions, and Post-Vaccination Side Effects among Rheumatic Disease Patients in Kuwait
Source: Vaccines (Basel). 2023 Mar 15;11(3):666. doi: 10.3390/vaccines11030666 (PMC10054305; doi:10.3390/vaccines11030666)
Supplement: Supplementary file 1 [file vaccines-11-00666-s001.zip › vaccines-2239066-supplementary/Supplementary file, English.pdf]

Dear Patient,

We hope this message finds you well and sound.
Based on the vision and perspective of the health responsibility of the Kuwait Association of Rheumatologists (KAR), we provide you with this questionnaire which highlights receiving the COVID-19 vaccine, through which we aim to:

- ✓ Estimate the relative numbers of patients who received COVID-19 vaccine among the patients with rheumatic diseases.
- ✓ Study the short-term symptoms post-vaccination, and if there is any flare of rheumatic disease.
- ✓ Highlight the real reasons why people refuse to get the vaccine (most common causes for non-vaccination).

We wish everyone safety, and thank you for your participation in filling out this questionnaire.

Hand in hand, we will fight the pandemic.

Regards,
Kuwait Association of Rheumatologists (KAR)

This English target text is a true translation of the Arabic source text. Issued on June 17, 2021 by PHARMA-MED for medical translation services, A.R.E.
[orders@pharmamed-eg.com]
All rights reserved

PHARMA MED Accreditation No.11271

ISO 17100 LICS Language Industry Certification System

Eligibility for taking part in the COVID-19 vaccine-related questionnaire

How did you know about this questionnaire?

- ❑ Rheumatologist
- ❑ Nurse at the infusion room
- ❑ Social media
- ❑ Drug Company
- ❑ A Friend/Relative
- ❑ Other

Eligibility for taking part in the COVID-19 vaccine-related questionnaire:

- ❑ You have a diagnosis of a rheumatic disease
- ❑ You are 21 years old or older.

Note: You are eligible to complete the questionnaire whether or not you have received the COVID-19 vaccine.

Consent

By participating, I confirm that I am 21 years of age at least and consent for the use of the information I provide in the analysis conducted by the Kuwait Association of Rheumatologists.

I understand that my data will be anonymous, and that I will not be able to be personally identified from my responses today.

I agree with the Kuwait Association of Rheumatologists to share the questionnaire data (without disclosing my identity) for purposes related to community awareness, education, and scientific research.

- ❑ Confirm
- ❑ Exit Questionnaire

Skip To: End questionnaire if the participant check "exit questionnaire"

This English target text is a true translation of the Arabic source text. Issued on June 17, 2021 by PHARMA-MED for medical translation services, A.R.E.
[orders@pharmamed-eg.com]
All rights reserved

1. Demographic characters

1.1. In what year were you born?

Skip To: End of questionnaire if condition: In what year were you born? Before or equal 2004, Skip To: End questionnaire.

1.2. What is your gender?

❑ Female

❑ Male

1.3. What is your nationality?

Drop list including all countries

1.4. In what country do you live?

Drop list including all countries

1.5. Who is your treating rheumatologist for your condition?

1.6. What is your educational level?

❑ Primary school certificate

❑ Middle school certificate

❑ High school certificate

❑ Vocational high school certificate

❑ Bachelor's degree

❑ Master's degree

❑ PhD degree

❑ Other (............)

1.7. Are you a healthcare provider?

❑ Yes

❑ No

This English target text is a true translation of the Arabic source text. Issued on June 17, 2021 by PHARMA-MED for medical translation services, A.R.E.
[orders@pharmamed-eg.com]
All rights reserved

1.8. What is your professional or employment status?

- ❑ Employed
- ❑ Not employed
- ❑ Retired
- ❑ Student
- ❑ Other (please specify)

1.9. What is your marital status?

- ❑ Single
- ❑ Married
- ❑ Divorced/Widow

This English target text is a true translation of the Arabic source text. Issued on June 17, 2021 by PHARMA-MED for medical translation services, A.R.E.
[orders@pharmamed-eg.com]
All rights reserved

2. Medical Situation

2.1. Do you smoke?

❑ Yes - I am smoke currently
❑ No - I used to smoke, but I gave up smoking
❑ No - I have never smoked

2.2. What disease of these have you been diagnosed with? [Select one option]

❑ Rheumatoid arthritis.
❑ Systemic lupus
❑ Systemic sclerosis
❑ Ankylosing spondylitis
❑ Psoriatic Arthritis
❑ Inflammation of the blood vessels (vasculitis)
❑ Dermatomyositis/Polymyositis
❑ Behçet disease
❑ Gout/pseudogout
❑ Sjögren's syndrome
❑ Reactive arthritis
❑ Enteropathic arthritis
❑ Connective tissue disease
❑ Juvenile arthritis
❑ Fibromyalgia
❑ Undifferentiated arthritis
❑ Still's disease (inflammatory disease)
❑ I was not diagnosed with a rheumatologic disease
❑ Not sure

Other [please specify] ....

Skip To: End of questionnaire if you have been diagnosed with the following? = I was not diagnosed with a rheumatic disease

All rights reserved

2.3. What approximate year were you diagnosed with this rheumatic condition?
For instance: *1996*

2.4. Which of the following medications have you taken during the past three months for the rheumatic disease? [Select all that apply]

- ❑ Abatacept (orencia)
- ❑ Adalimumab (Humira)
- ❑ Aprimilast (Otezla)
- ❑ Azathioprine (Imuran)
- ❑ Baricitinib (Ollumiant)
- ❑ Belimumab (Benlista)
- ❑ Certizolumab (Cimzia)
- ❑ Chloroquine (Dafoquine)
- ❑ Corticosteroids
- ❑ Cyclophosphamide
- ❑ Etanercept (Enbril)
- ❑ Golimumab (Simponi)
- ❑ Hydroxychloroquine (Plaquenil)
- ❑ Infliximab (Remicade)
- ❑ Leflunomide (Arava)
- ❑ Methotrexate
- ❑ Mycophenolate mofetil (cellcept/Myfortic))
- ❑ Rituximab (MabThera)
- ❑ Secukinumab (cosentyx)
- ❑ Sulfasalazine (salazopyrin)
- ❑ Tacrolimus (Prograf)
- ❑ Tocilizumab (Actemra)
- ❑ Tofacitinib (Xeljenz)
- ❑ UStekinumab (Stelara)
- ❑ I do not take any medication
- ❑ Not sure
- ❑ Other [please specify] ....

This English target text is a true translation of the Arabic source text. Issued on June 17, 2021 by PHARMA-MED for medical translation services, A.R.E.
[orders@pharmamed-eg.com]
All rights reserved

PHARMA MED Accreditation No.11271

ISO 17100 LICS Language Industry Certification System

2.5. How best would you describe your rheumatic disease in the previous three months?

- ❑ Inactive
- ❑ Mild to moderately active
- ❑ Severely active

2.6. Do you have any other chronic diseases?

- ❑ Diabetes.
- ❑ Heart disease
- ❑ High Blood Pressure
- ❑ Lung disease
- ❑ Obesity
- ❑ Kidney disease
- ❑ Cancer
- ❑ Immunodeficiency, including HIV
- ❑ None
- ❑ Other [please specify]

This English target text is a true translation of the Arabic source text. Issued on June 17, 2021 by PHARMA-MED for medical translation services, A.R.E.
[orders@pharmamed-eg.com]
All rights reserved
PHARMA MED Accreditation No.11271
ISO 17100 LICS Language Industry Certification System

3. COVID-19-related situation

3.1. Have you received a positive swab result confirming that you are infected with COVID-19?

❑ Yes

❑ No

If *'No'*

3.1.1. Have you been quarantined during the period from 2019 to 2021?

❑ Yes
❑ No

If 'Yes'

3.1.1.1. What was the reason?

❑ *Traveling*

❑ *In contact with a person infected with the virus*

3.1.2. How concerned are you to get infected by the COVID-19?

❑ Not concerned
❑ Mildly concerned
❑ Moderately concerned
❑ Deeply concerned
❑ Extremely concerned

3.1.3. How concerned are you to get infected with severe COVID-19?

❑ Not concerned
❑ Mildly concerned
❑ Moderately concerned
❑ Deeply concerned
❑ Extremely concerned

If 'Yes'

3.2.1. What was the date of positive results? [Insert calendar here]

3.2.2. How things have turned out? [Select one option]

❑ I was not hospitalized

❑ I was hospitalized, but I was not given supplemental oxygen

❑ I was hospitalized with supplemental oxygen

❑ I was hospitalized with the ICU admission

This English target text is a true translation of the Arabic source text. Issued on June 17, 2021 by PHARMA-MED for medical translation services, A.R.E.
[orders@pharmamed-eg.com]
All rights reserved

3.3. Since the beginning of the pandemic, has someone around you (friends, colleagues, relatives) been infected with a severe COVID (leading to hospitalization, ICU, or death)?

❑ Yes

❑ No

All rights reserved

PHARMA MED
Accreditation No.11271

ISO 17100 LICS®
Language Industry Certification System

4. Vaccine-related situation

4.1. Do you have allergy to any vaccine?

❑ Yes
❑ No
❑ Not Sure/I don't remember

4.2. Have you ever refused to take a recommended vaccine or refused to give it to one of your children?

❑ Yes
❑ No
❑ Not Sure/I don't remember

4.3. Did you receive the flu vaccine (to prevent colds) in 2019?

❑ Yes
❑ No
❑ Not Sure/I don't remember

4.4. Did you receive the flu vaccine (to prevent colds) in 2020?

❑ Yes
❑ No
❑ Not Sure/I don't remember

4.5. Did you receive the Pneumococci vaccine?

❑ Yes
❑ No
❑ Not Sure/I don't remember

This English target text is a true translation of the Arabic source text. Issued on June 17, 2021 by PHARMA-MED for medical translation services, A.R.E.
[orders@pharmamed-eg.com]
All rights reserved
PHARMA MED Accreditation No.11271
ISO 17100 LICS Language Industry Certification System

5. COVID-19 Vaccine

5.1. Have you consulted your doctor about the COVID-19 vaccine?

❑ Yes
❑ No
❑ Not Sure/I don't remember

5.2. Have you been advised by your doctor not to receive the vaccine?

❑ Yes
❑ No
❑ Not Sure/I don't remember

5.3. Did you read the online advice sheet provided by the Kuwait Association of Rheumatologists about COVID-19 vaccine?

❑ Yes
❑ No
❑ Not Sure/I don't remember

5.4. What is your source of information regarding the vaccine? [Select all that apply]

❑ I have no information
❑ News media (TV/radio/newspaper)
❑ Social media
❑ Public health advice provided by the government
❑ Friends/Family
❑ Medical publications
❑ Your doctor(s) or other health professionals
❑ Patient or professional organization(s)
❑ Other [please specify] ....

This English target text is a true translation of the Arabic source text. Issued on June 17, 2021 by PHARMA-MED for medical translation services, A.R.E.
[orders@pharmamed-eg.com]
All rights reserved
PHARMA MED Accreditation No.11271
ISO 17100 LICS Language Industry Certification System

5.5 Did you receive the COVID-19 vaccine?

❑ Yes

❑ No

If 'Yes':

5.5.1. What was the reason for receiving the COVID-19 vaccine?

❑ I am afraid of getting infected/severe infection with COVID-19

❑ I am afraid of death from COVID-19

❑ I want to protect my family from COVID-19

❑ Because receiving the vaccine will reduce the financial burden incurred due to the pandemic

❑ I believe that the vaccine is the only way that can help us return to how life was before COVID-19

❑ Because I generally trust information and instructions issued by the government

5.5.2. Which vaccine did you receive?

❑ Pfizer/BioNTech

❑ AstraZeneca/Oxford

❑ Moderna

❑ Johnson & Johnson

❑ Sputnik V

❑ Sinovac

❑ Other [please specify] ....

5.5.3. When was the date of the first dose?

[Insert Calendar here]

5.5.4. When was the date of the second dose?

(Leave it blank if you have not received it yet or not applicable)

[Insert Calendar here]

5.5.5. Have you stopped taking your medications or have adjustments been made?

❑ Yes

❑ No

❑ Not Sure/I don't remember

5.5.6. If 'Yes', please specify [………..]

All rights reserved

5.5.7. Did you experience and side effects after receiving your COVID-19 vaccine?

- ❑ Anaphylaxis
- ❑ Blood Clots
- ❑ Blood pressure disorders
- ❑ Abnormalities in the blood glucose level
- ❑ Body ache/pain
- ❑ Rash in the face and body
- ❑ Fatigue
- ❑ Fever
- ❑ Headache
- ❑ Joint swelling
- ❑ Bruise/local rash at the injection site
- ❑ Nausea/vomiting
- ❑ Neurological symptoms
- ❑ Pain at the injection site
- ❑ Difficulty breathing
- ❑ Flare of rheumatic disease worsening (require to see a doctor)
- ❑ Swollen lymph nodes
- ❑ None.
- ❑ Other [please specify] ....

5.5.8. How long has the side effects lasted for?

- ❑ From 1 to 5 days
- ❑ From 5 to 10 days
- ❑ From 10 to 14 days
- ❑ More than 14 days

5.5.9. Do you think receiving the COVID-19 vaccine should be mandatory?

- ❑ Yes
- ❑ No

This English target text is a true translation of the Arabic source text. Issued on June 17, 2021 by PHARMA-MED for medical translation services, A.R.E.
[orders@pharmamed-eg.com]
All rights reserved

If 'No':

5.5.10. You did not receive COVID-19 vaccine because of:

❑ I am waiting for the vaccine

❑ I am against receiving COVID-19 vaccines

❑ I have requested an appointment, but has been rejected by the Ministry of Health (due to anaphylaxis, pregnancy, COVID-19 within the previous 90 days, age under 16, or active infection)

Skip To "section 6" If you did not receive COVID-19 vaccine because of = I am against the campaign for receiving the COVID-19 vaccine

This English target text is a true translation of the Arabic source text. Issued on June 17, 2021 by PHARMA-MED for medical translation services, A.R.E.
[orders@pharmamed-eg.com]
All rights reserved
PHARMA MED Accreditation No.11271
ISO 17100 LICS Language Industry Certification System

6. Opposition to receiving the vaccine

Only for subjects whose answer to the question "You did not receive COVID-19 vaccine because of" = I am against the campaign for receiving the COVID-19 vaccine

6.1. Which phrase best describes you?

❑ I am against receiving the vaccine in general

❑ I am against receiving the COVID-19 vaccine because I am not sure of its efficacy

❑ I am against receiving the COVID-19 vaccine due to concerns about its side effects

❑ I am against receiving the COVID-19 vaccine because there is not enough data, and because patients with rheumatic disease have been excluded from the studies

❑ I believe that I may get infected with COVID-19 after receiving the vaccine

❑ I am against receiving the COVID-19 vaccine because I am afraid it will worsen my disease or interfere with my treatment

❑ I am against receiving the COVID-19 vaccine because I believe it is recommended for economic reasons supported by pharmaceutical companies

❑ The reason for refusing to receive the vaccine is trypanophobia

❑ The reason for refusing vaccine is because I have previously had a vaccine anaphylaxis

6.2. Would you like to receive the vaccine if more data is available about safety and efficacy of the different vaccines?

❑ Yes

❑ No

❑ I do not know

6.3. Has any family members (first degree) got vaccinated?

❑ Yes

❑ No

❑ I do not know

6.4. Do you advice any of your family members or relatives not to receive COVID-19 vaccine?

❑ Yes

❑ No

This English target text is a true translation of the Arabic source text. Issued on June 17, 2021 by PHARMA-MED for medical translation services, A.R.E.
[orders@pharmamed-eg.com]
All rights reserved

6.5. If COVID-19 vaccine becomes mandatory, will you receive it?

- ❑ Yes
- ❑ No
- ❑ I do not know

6.6. If you are invited to an awareness event about the importance of COVID-19 vaccine, will you attend?

- ❑ Yes
- ❑ No

All rights reserved
PHARMA MED
Accreditation No.11271
ISO 17100 LICS Language Industry Certification System
